# Impaired embryo development potential associated with thyroid autoimmunity in euthyroid infertile women with diminished ovarian reserve

**DOI:** 10.3389/fendo.2024.1376179

**Published:** 2024-06-14

**Authors:** Yongjie Zhang, Yuchao Zhang, Zhuolun Su, Bingnan Ren, Shuang Yu, Wenjing Li, Ninghua Xu, Hua Lou

**Affiliations:** Reproductive Center, The Third Affiliated Hospital of Zhengzhou University, Zhengzhou, China

**Keywords:** thyroid autoimmunity, diminished ovarian reserve, thyroid antibodies, number of oocytes retrieved, fertilization rate, Embryo quality

## Abstract

**Purpose:**

The aim of this study was to evaluate the associations of thyroid autoimmunity (TAI) with the number of oocytes retrieved (NOR), fertilization rate (FR), and embryo quality (EQ) in euthyroid women with infertility and diminished ovarian reserve (DOR).

**Methods:**

This retrospective cohort study involved 1,172 euthyroid women aged 20–40 years with infertility and DOR who underwent an oocyte retrieval cycle. TAI was diagnosed in the presence of serum thyroperoxidase antibody (TPOAb) concentrations higher than 34 IU/ml and/or serum thyroglobulin antibody (TgAb) concentrations exceeding 115.0 IU/ml. Among these women, 147 patients with TAI were classified as the TAI-positive group, while 1,025 patients without TAI were classified as the TAI-negative group. Using generalized linear models (GLMs) adjusted for confounding factors, we evaluated the associations of TAI and the serum TPOAb and TgAb concentrations and NOR, FR, and EQ in this study’s subjects. The TPOAb and TGAb values were subjected to log10 transformation to reduce skewness. Logistic regression models were used to estimate the effects of TPOAb and TgAb concentrations on the probabilities of achieving a high NOR (≥7) and high FR (>60%).

**Results:**

For the whole study population, women with TAI had a significantly lower NOR and poorer EQ than women without TAI (*P* < 0.001 for both). Interestingly, in the TSH ≤2.5 subgroup, the TAI-positive group also had a significantly lower NOR and poorer EQ than the TAI-negative group (*P* < 0.001 for both). Furthermore, negative associations were observed between log10(TPOAb) concentrations and NOR and the number of high-quality embryos and available embryos (*P* < 0.05 for all). The log10(TgAb) concentrations were inversely associated with NOR and the number of high-quality embryos (*P* < 0.05 for all). In the regression analysis, the log10(TPOAb) concentrations had lower probabilities of achieving a high NOR [adjusted odds ratio (aOR): 0.56; 95% confidence interval (95% CI) 0.37, 0.85; *P* = 0.007].

**Conclusions:**

TAI and higher TPOAb and TgAb concentrations were shown to be associated with reductions in the NOR and EQ in the study population. Our findings provide further evidence to support systematic screening and treatment for TAI in euthyroid women with infertility and DOR.

## Introduction

The successful progression of various intricate processes, including female reproduction, embryonic development, maternal–fetal recognition, and the establishment of placental circulation, is essential for ensuring reproductive success. Immune regulation plays a significant role in these processes (1, 2). An imbalance in self-immunity and immunity toward the same species may be linked to adverse outcomes such as diminished ovarian reserve (DOR) and pregnancy loss (3). Thyroid autoimmunity (TAI), which is characterized by the presence of serum autoantibodies against thyroperoxidase (TPOAb) or thyroglobulin (TgAb), is the most common autoimmune disorder and also the leading cause of thyroid dysfunction among women of reproductive age (4). The relationship between TAI and pregnancy outcomes following *in-vitro* fertilization (IVF)/intracytoplasmic sperm injection (ICSI) cycles has been widely studied by researchers for many years. Studies have shown that in euthyroid patients with infertility, TAI is associated with adverse assisted reproductive technology (ART) outcomes, including a higher miscarriage rate and a lower live birth rate (LBR) (5, 6). The exact pathophysiological mechanism underlying this association is currently unknown. It was hypothesized that among women with infertility, TAI might impact outcomes such as the number of retrieved oocytes (NOR), embryo quality (EQ), and fertilization rate (FR) during the earliest stages following the use of ART (7–10). Nearly all previous relevant studies have focused on the whole population of patients with infertility. DOR is a major cause of female infertility during their reproductive years. The causes of DOR are varied and complex, potentially leading to premature ovarian failure. Moreover, possible correlations between DOR and decreases in oocyte quality and EQ were reported (11). The prevalence of TAI was found to be significantly elevated in patients with premature ovarian insufficiency (POI), with prevalence rates ranging from 14% to 27% (4, 12). This rate is in stark contrast to the TAI prevalence rates of 8%–18% observed in the general population (13–15). If thyroid function and/or TAI affect the *in-vitro* outcomes of ART, this impact should be particularly evident in women with DOR, from whom a limited number of oocytes can be retrieved during *in-vitro* fertilization. Given the lower probability of successful conception in women with DOR in natural conception and ART treatments, studying this population not only has important clinical value but also can provide more targeted guidance and treatment recommendations. Currently, only one study has investigated the influence of TAI on EQ in euthyroid patients with infertility and low functional ovarian reserve (16). It is unclear whether TAI exacerbates the earliest stage *in-vitro* outcomes of ART and pregnant outcomes among euthyroid women with infertility and DOR. Given the complexity of embryonic development, the potential regulatory effects of age, serum thyroid-stimulating hormone (TSH) concentrations, and the ovulation process should not be overlooked (17). Therefore, better research designs are needed to investigate the association between TAI and embryonic development. In addition, previous studies have mainly focused on qualitative analysis of thyroid autoantibodies, namely, positive and negative analyses (7, 14, 16, 18). Less attention has been given to the concentrations of these autoantibodies. Therefore, it remains unclear whether the concentrations of thyroid autoantibodies are associated with *in-vitro* outcomes of ART in euthyroid women with DOR. With this study, we aimed to study the relationships of TAI and elevated thyroid antibody concentrations with *in-vitro* outcomes of ART and pregnant outcomes in euthyroid women with DOR.

## Material and methods

### Study design and population

The data of patients who underwent an oocyte retrieval cycle at the Center of Reproductive Medicine, the Third Affiliated Hospital of Zhengzhou University, from January 2020 to October 2023 were retrospectively screened. A total of 3,233 infertile women aged 20–40 years with DOR were considered potential candidates for inclusion in this study. DOR was defined by any of the following criteria: regular or irregular menses, accompanied by basal follicle-stimulating hormone (FSH) concentration greater than or equal to 10 IU/L at an interval of more than 4 weeks apart; an anti-Müllerian hormone (AMH) concentration less than 1.1 ng/ml; or bilateral antral follicle count (AFC) less than 7. These measurements should be taken on days 2–4 of the menstrual cycle (19). The following exclusion criteria were applied: i) the absence of TPO-Ab, TgAb, or thyroid function indicators including TSH, free triiodothyronine (FT3), and free thyroxine (FT4); ii) thyroid function outside the reference range (0.27–4.2 mIU/L for TSH, 3.1–6.8 pmol/L for FT3, and 12.0–22.0 pmol/L for FT4); iii) a history of thyroid disease, levothyroxine (LT4) or antithyroid medication treatment, or thyroid surgery; iv) a history of recurrent spontaneous abortion; v) chromosomal abnormalities; and vi) other autoimmune disorders, such as systemic lupus erythematosus, rheumatoid arthritis, or Sjögren’s syndrome.

**Figure 1:**
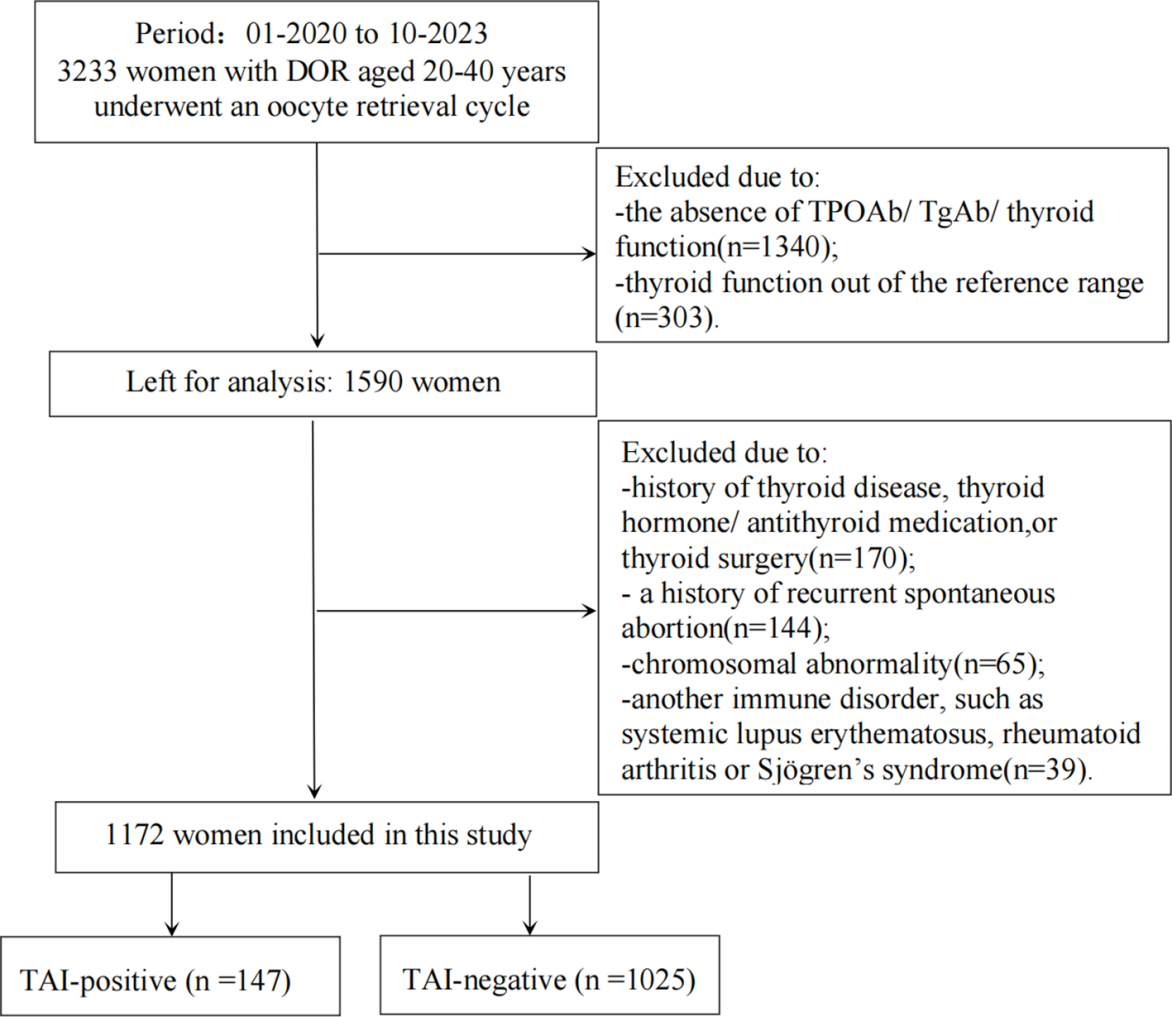
Flowchart depicting the participant screening in the study. DOR, diminished ovarian reserve; TSH, thyrotropin; FT3, free triiodothyronine; FT4, free thyroxine; TPOAb, thyroperoxidase antibody; TgAb, thyroglobulin antibody; TAI, thyroid autoimmunity.

As shown in **Figure 1**, among the 1,172 patients finally included as subjects in the study, 147 patients with TAI were classified as the TAI-positive group, while 1,025 patients without TAI were classified as the TAI-negative group. TAI was diagnosed in the presence of serum TPOAb concentrations higher than 34 IU/ml and/or serum TgAb concentrations exceeding 115.0 IU/ml. All data, including the subjects’ baseline characteristics and outcome indicators, were systematically recorded for both groups.

### Biochemical measures and ultrasonography

On the second or third day of the menstrual cycle, vaginal ultrasound was used to measure both the number and size of bilateral AFC. Venous blood samples were also collected from all subjects on the second or third day of a menstrual cycle to measure the concentrations of hormones, namely, FSH, AMH, TSH, FT3, and FT4, and thyroid autoantibodies, namely, TgAb and TPOAb. The samples were centrifuged at 3,000 rpm for 10 min after half an hour of sampling. The serum on the upper layer was used for analysis. The resulting serum samples were measured using an electrochemical luminescence (ECLIA) on a Cobas 8000 (Roche Diagnostics, Germany). The following reference ranges for TSH, FT3, and FT4 concentrations were used by our reproductive center: TSH, 0.27–4.2 mIU/L; FT3, 3.1–6.8 pmol/L; and FT4, 12.0–22.0 pmol/L. For thyroid antibodies, the following reference thresholds were used: TPOAb, 34 IU/ml, and TgAb, 115 IU/ml. The TPOAb or TgAb concentrations exceeding these thresholds were considered to indicate the presence of TAI.

### Ovarian stimulation

The women included in the current study were subjected to a standardized ovarian stimulation regimen that encompassed gonadotropin-releasing hormone agonist (GnRH-a) protocols, gonadotropin-releasing hormone antagonist (GnRH-ant) protocols, and mild stimulation protocols (20). GnRH-a is used to promote synchronization of follicular development (21). A tailored ovarian stimulation regimen was designed, taking into account each patient’s age, body mass index (BMI), and ovarian reserve. Specifically, for women with DOR, milder stimulation protocols and GnRH-ant protocols were more commonly used (22). Regardless of the protocol employed, routine monitoring of serum hormone levels, such as luteinizing hormone (LH), estradiol (E2), and progesterone (P), was conducted during the stimulation process. Concurrently, follicular development was observed via transvaginal ultrasonography.

### Fertilization and embryo culture

Once two or three dominant follicles had reached a diameter of 18 mm or more, human chorionic gonadotropin was administered either alone or in combination with a GnRH-a via intramuscular injection. Oocyte retrieval under transvaginal ultrasound guidance was scheduled 36–38 h later. The choice of a conventional IVF (cIVF) or ICSI was based on the semen analysis results obtained on the day of the procedure. In cIVF, sperm was prepared using the Markler plate method to a concentration of (5–10) × −10^6^/ml. Subsequently, 40 to 60 μl of sperm and the partner’s oocytes were added to a culture dish. The sperm and oocytes were then co-cultured for insemination in an incubator for 4–6 h or overnight to remove granulosa cells. For ICSI, a single sperm was injected into the oocyte after 38–40 h of triggering. Fertilization and embryo assessment were performed by experienced embryologists. The quality of cleavage embryos and blastocysts was evaluated based on the Istanbul consensus and as described in previous studies (20, 23). Specifically, for cleavage embryos, the quality score was mainly based on the number of blastomeres, blastomere size, and fragmentation. Cleavage-stage embryos of good quality were defined as having a uniform number of blastomeres between 6 and 10, with a fragmentation rate of less than 20%. The quality of blastocysts was mainly scored based on the developmental stage (24–27), inner cell mass quality (A, B, C), and trophectoderm quality (A, B, C). Blastocysts with a developmental stage greater than 3 were considered to have good quality; both inner cell mass quality and trophectoderm quality are superior to grade C. Women underwent embryo transfer with either cleavage embryos or a blastocyst in the fresh cycles. For cleavage-stage embryos, a maximum of two embryos could be selected for transfer. For blastocyst, only one blastocyst was chosen for transfer. The selection of the transfer strategy is based on the specific condition of the patient.

### Data analysis

Continuous data adhering to a normal distribution are reported as the mean [standard deviation (SD)], and Student’s *t*-test was used to perform between-group comparisons. The normality of data distribution was evaluated using the Kolmogorov–Smirnov test. Continuous data that do not fit a normal distribution are reported as the median [interquartile range (IQR)], and the Mann–Whitney *U* test was used for group comparisons. Categorical data are presented as the count (percentage), and the chi-square test or Fisher’s exact test was used for analysis. The TPOAb and TgAb concentrations were subjected to log10 transformation in statistical analyses to reduce skewness. The base-10 logarithm-adjusted concentrations of TPOAb and TgAb were represented as log10(TPOAb) and log10(TgAb), respectively. Poisson regression models using the generalized linear model (GLM) approach were used to evaluate the association of TAI or the concentrations of log10(TPOAb) and log10(TgAb) with the total number of oocytes retrieved, available embryos, and high-quality embryos (28). Ratio data, including FR and blastocyst rate, were assessed by negative binomial regression models using the GLM approach. The association of log10(TPOAb) and log10(TgAb) concentrations with a high NOR and high FR was analyzed using binary logistic regression models. The cutoffs for a high NOR (≥7) and high FR (>60%) were derived from relevant literature within this discipline (29, 30). Binary logistic regression models were used to analyze the clinical pregnancy rate (CPR), miscarriage rate (MR), and LBR. In the above models, we utilized prior knowledge about medical relevance and descriptive statistics of our study population to assess confounding factors, using a directed acyclic graph (31). The variables considered as potential confounders included factors previously associated with reproductive outcomes in this study and other studies, as well as factors associated with TAI exposure and reproductive outcomes. The models used to analyze the impact of TAI or TPOAb and TgAb concentrations on NOR were adjusted for several baseline characteristics, including TSH, FT3, FT4, female age, BMI, total Gn dose, GnRH-a used, and AFC. The models used to analyze the impact of TAI or TPOAb and TgAb concentrations on FR, blastocyst rate, EQ, and pregnancy outcomes were further adjusted for the ART type used and male age. Data analyses were performed using SPSS 26.0 (SPSS, Inc., Chicago, IL, USA). All the significance levels of the analyses are two-tailed *P <*0.05.

## Results

### Differences in baseline characteristics between the groups

As shown in **Table 1**, the mean age of the TAI-positive group was significantly higher than that of the TAI-negative group (34.69 ± 3.71 vs. 33.84 ± 4.05 years, *P* = 0.016). However, the percentage of patients older than 35 was comparable between the two groups [62 (42.18%) vs. 375 (36.59%), *P* > 0.05]. The TAI-positive group had a higher likelihood of having a TSH concentration exceeding 2.5 mIU/L than the TAI-negative group [67 (45.58%) vs. 362 (35.32%), respectively, *P* = 0.016] but had comparable mean TSH (2.32 ± 0.95 vs. 2.27 ± 0.85 mIU/L, respectively, *P* = 0.462) and mean FT4 levels (16.46 ± 2.07 vs. 16.52 ± 2.04 pmol/L, respectively, *P* = 0.753). The mean FT3 concentrations of the TAI-positive group were significantly lower than those of the TAI-negative group (4.74 ± 0.56 vs. 4.85 ± 0.55 pmol/L, respectively, *P* = 0.021). In assisted reproductive cycles, various parameters, including the type of infertility, mean male age, ART type used, GnRH-a used, total Gn dose, ovarian reserve parameters (basal FSH and AMH concentrations), and bilateral AFC, were comparable between the two groups (*P* > 0.05 for all).

**Table 1:** Baseline characteristics of the included subjects.

Characteristics	TAI-positive (*n* = 147)	TAI-negative (*n* = 1,025)	*P*
Female age, mean (SD), years	34.69 (3.71)	33.84 (4.05)	0.016
Age >35 years, *n* (%)	62 (42.18)	375 (36.59)	0.190
Male age, mean (SD), years	34.77 (5.23)	34.44 (5.05)	0.468
Female BMI, mean (SD), kg/m^2^	23.78 (3.30)	23.74 (3.45)	0.891
TSH, mean (SD), mIU/L	2.32 (0.95)	2.27 (0.85)	0.462
TSH >2.5 mIU/L, *n* (%)	67 (45.58)	362 (35.32)	0.016
FT3, mean (SD), pmol/L	4.74 (0.56)	4.85 (0.55)	0.021
FT4, mean (SD), pmol/L	16.46 (2.07)	16.52 (2.04)	0.753
TPOAb, median (IQR), mIU/L	49.86 (12.64, 158.95)	10.13 (7.82, 13.33)	<0.001
TgAb, median (IQR), mIU/L	255.70 (129.30, 414.55)	15.71 (13.52, 18.43)	<0.001
AMH, median (IQR), ng/ml	0.75 (0.47, 0.97)	0.72 (0.44, 0.95)	0.718
Bilateral AFC, mean (SD)	7.22 (3.54)	7.09 (3.68)	0.696
Baseline FSH, mean (SD), IU/L	9.71 (6.52)	9.62 (5.03)	0.856
Type of infertility, *n* (%)			0.882
Primary	56 (38.10)	384 (37.46)	
Secondary	91 (61.90)	641 (62.54)	
GnRH-a used, *n* (%)	34 (23.13)	233 (22.73)	0.914
Total Gn dose, mean (SD)	2,769.81 (1,338.53)	2,921.92 (1,153.47)	0.142
ART type used, *n* (%)			0.364
IVF	110 (74.83)	730 (71.22)	
ICSI	37 (25.17)	295 (28.78)	

TAI, thyroid autoimmunity; BMI, body mass index; TSH, thyrotropin; FT3, free triiodothyronine; FT4, free thyroxine; TPOAb, thyroperoxidase antibody; TgAb, thyroglobulin antibody; AFC, antral follicle count; AMH, anti-Müllerian hormone; FSH, follicle-stimulating hormone; E2, estradiol; GnRH-a, gonadotropin-releasing hormone agonist; Gn, gonadotropin; ART, assisted reproductive technology; IQR, interquartile range; SD, standard deviation.

### Impact of TAI on the NOR, FR, and EQ

After adjustment for potential confounding variables, Poisson GLMs revealed significant negative associations between TAI and the NOR [Beta: −0.12 (95% confidence interval, 95% CI: −0.20, −0.04); *P* = 0.003] and the numbers of available embryos [Beta: −0.14 (95% CI: −0.25, −0.04); *P* = 0.007] and high-quality embryos [Beta: −0.22 (95% CI: −0.36, −0.08); *P* = 0.002]. After exponentiation, compared with the TAI-negative group, the average NOR and the numbers of available embryos and high-quality embryos in the TAI-positive group decreased by 12% (95% CI: −11.9%, −4.2%; *P* = 0.003), 14.3% (95% CI: −24.6%, −4.0%; *P* =0.007), and 22.3% (95% CI: −36.3%, −8.2%; *P* = 0.002), respectively. The FR and blastocyst rate were not significantly different between the TAI-positive and TAI-negative groups (*P* > 0.05 for both) (**Table 2**). Of note, in the subgroup of women with TSH concentrations ≤2.5 mIU/L, after adjustment for confounders, TAI positivity was negatively associated with the NOR and the numbers of high-quality embryos and available embryos (*P* < 0.05 for all). However, in the subgroup of women with TSH concentrations >2.5 mIU/L, no differences in these outcomes were observed between the two groups (*P* > 0.05, for all) (**Table 3**). The CPR and LBR were not significantly different between the two groups (*P* > 0.05 for both) (**Supplementary Table S1**). However, the MR in the TAI-positive group was significantly higher than that in the TAI-negative group [adjusted odds ratio (aOR): 4.37; 95% CI: 1.25, 15.25; *P* = 0.021] (**Supplementary Table S1**).

**Table 2:** Generalized linear analysis of the number of oocytes retrieved, fertilization rate, and embryo quality according to TAI status.

Outcomes	TAI-positive (*n* = 147)	TAI-negative (*n* = 1,025)	Beta (95% CI)	*P*
No. of oocytes retrieved, mean (SD)^a^	4.78 (3.16)	5.39 (3.18)	−0.12 (−0.20, −0.04)	0.003
No. of embryos available, mean (SD)^b^	2.78 (2.29)	3.20 (2.33)	−0.14 (−0.25, −0.04)	0.007
No. of high-quality embryos, mean (SD)^b^	1.48 (1.69)	1.84 (1.82)	−0.22 (−0.36, −0.08)	0.002
Fertilization rate, *n* (%)^b^	478 (68.0)	3,878 (70.2)	−0.16 (−0.61, −0.29)	0.489
Blastocyst rate, *n* (%)^b^	149 (53.91)	1,236 (56.15)	−0.02 (−0.10, 0.07)	0.658

TAI, thyroid autoimmunity; BMI, body mass index; TSH, thyrotropin; FT3, free triiodothyronine; FT4, free thyroxine; Gn, gonadotropin; GnRH-a, gonadotropin-releasing hormone agonist; AFC, antral follicle count; ART, assisted reproductive technology; CI, confidence interval; SD, standard deviation.

a Adjusted for maternal age; maternal BMI; TSH, FT3, and FT4 concentrations; total Gn dose; GnRH-a used; type of infertility; and AFC.

b Adjusted for maternal age; maternal BMI; TSH, FT3, and FT4 concentrations; total Gn dose; GnRH-a used; type of infertility; AFC; ART type used; and male age.

**Table 3:** Association between thyroid autoimmunity and embryo quality at different TSH concentrations.

Outcomes	TSH ≤2.5 mIU/L				TSH >2.5 mIU/L			
	TAI-positive (*n* = 80)	TAI-negative (*n* = 663)	Beta (95% CI)	*P*	TAI-positive (*n* = 67)	TAI-negative (*n* = 362)	Beta (95% CI)	*P*
No. of oocytes retrieved, mean (SD)^a^	4.56 (2.52)	5.47 (3.21)	−0.18 (−0.29, −0.07)	0.001	5.04 (3.80)	5.25 (3.13)	−0.04 (−0.16, 0.08)	0.502
No. of embryos available, mean (SD)^b^	2.58 (1.77)	3.3 (2.43)	−0.25 (−0.39, −0.10)	0.001	3.01 (2.79)	3.02 (2.12)	−0.00 (−0.15, 0.15)	0.965
No. of high-quality embryos, mean (SD)^b^	1.35 (1.48)	1.90 (1.83)	−0.34 (−0.54, −0.14)	0.001	1.63 (1.92)	1.74 (1.80)	−0.07 (−0.28, 0.13)	0.489
Fertilization rate, *n* (%)^b^	242 (66.19)	2,564 (71.48)	−0.12 (−0.57, 0.33)	0.602	236 (70.78)	1,314 (66.35)	−0.22 (−0.89, 0.46)	0.534
Blastocyst rate, *n* (%)^b^	67 (49.93)	842 (56.99)	−0.27 (−0.98, 0.45)	0.469	82 (59.33)	394 (54.62)	0.14 (−0.66, 0.95)	0.726

TAI, thyroid autoimmunity; BMI, body mass index; TSH, thyrotropin; FT3, free triiodothyronine; FT4, free thyroxine; Gn, gonadotropin; GnRH-a, gonadotropin-releasing hormone agonist; AFC, antral follicle count; ART, assisted reproductive technology; CI, confidence interval; SD, standard deviation.

a Adjusted for maternal age; maternal BMI; TSH, FT3, and FT4 concentrations; total Gn dose; GnRH-a used; type of infertility; and AFC.

b Adjusted for maternal age; maternal BMI; TSH, FT3, and FT4 concentrations; total Gn dose; GnRH-a used; type of infertility; AFC; ART type used; and male age.

### Effect of antibody concentrations on the NOR and EQ

The concentrations of TPOAb and TgAb were log-transformed to base 10, represented as log10(TPOAb) and log10(TgAb), respectively. The higher concentrations of log10(TPOAb) were significantly associated with lower NOR [Beta: −0.11 (95% CI: −0.18, −0.05); *P* = 0.001] and the numbers of available embryos [Beta: −0.10 (95% CI: −0.19, −0.01); *P* = 0.026] and high-quality embryos [Beta: −0.22 (95% CI: −0.34, −0.10); *P* < 0.001) when using Poisson GLMs adjusted for confounding factors. After exponentiation, each 1-unit increase in the log10(TPOAb) corresponded to average decreases of 11.3% in the NOR (95% CI: −17.9%, −4.6%; *P* = 0.001), 9.9% in the number of available embryos (95% CI: −18.5%, −1.2%; *P* = 0.026), and 21.8% in the number of high-quality embryos (95% CI: −33.6%, −10.1%; *P* < 0.001). Similarly, as the log10(TgAb) concentrations increased, the NOR [Beta: −0.07 (95% CI: −0.12, −0.02); *P* = 0.008] and the number of high-quality embryos [Beta: −0.12 (95% CI: −0.22, −0.03); *P* = 0.011] also decreased. After exponentiation, each 1-unit increase in the log10(TgAb) was associated with average decreases of 7.2% in the NOR (95% CI: −12.5%, −1.8%; *P* = 0.008) and 12.0% in the number of high-quality embryos (95% CI: −21.2%, −2.7%; *P* = 0.011). Although not significant, each 1-unit increase in the log10(TgAb) was associated with an average decrease of 6.0% in the number of available embryos (95% CI: −12.9%, −0.9%; *P* = 0.086) (**Table 4**).

**Table 4:** Poisson regression analysis of the number of oocytes retrieved and embryo quality according to antibody concentrations.

Outcomes	NOR^a^		No. of available embryos^b^		No. of high-quality embryos^b^	
Variables	Beta (95% CI)	*P*	Beta (95% CI)	*P*	Beta (95% CI)	*P*
Log10(TPOAb)	−0.11 (−0.18, −0.05)	0.001	−0.10 (−0.19, −0.01)	0.026	−0.22 (−0.34, −0.10)	<0.001
Log10(TgAb)	−0.07 (−0.13, −0.02)	0.008	−0.06 (−0.13, −0.01)	0.086	−0.12 (−0.22, −0.03)	0.011

NOR, number of oocytes retrieved; TPOAb, thyroperoxidase antibody; TgAb, thyroglobulin antibody; BMI, body mass index; TSH, thyrotropin; FT3, free triiodothyronine; FT4, free thyroxine; Gn, gonadotropin; GnRH-a, gonadotropin-releasing hormone agonist; AFC, antral follicle count; ART, assisted reproductive technology; CI, confidence interval.

a Adjusted for maternal age; maternal BMI; TSH, FT3, and FT4 concentrations; total Gn dose; GnRH-a used; type of infertility; and AFC.

b Adjusted for maternal age; maternal BMI; TSH, FT3, and FT4 concentrations; total Gn dose; GnRH-a used; type of infertility; AFC; ART type used; and male age.

### Influence of antibody concentrations on a high NOR and high FR

The logistic regression analysis revealed that log10(TPOAb) concentrations were negatively associated with a high NOR [aOR: 0.56 (95% CI: 0.37, 0.85); *P* = 0.007]. However, the log10(TgAb) concentrations were not associated with a high NOR [aOR: 0.73 (95% CI: 0.53, 1.03); *P* = 0.063]. The log10(TPOAb) and the log10(TgAb) concentrations were both not found to be associated with a high FR [aOR: 0.93 (95% CI: 0.67, 1.29); *P* = 0.653; aOR: 0.99 (95% CI: 0.76, 1.29); *P* = 0.953] (**Table 5**).

**Table 5:** Logistic regression analysis of factors associated with the high number of oocytes retrieved and high fertilization rates.

Outcomes	High NOR (≥7)^a^		High FR (>60%)^b^	
Variables	aOR (95% CI)	*P*	aOR (95% CI)	*P*
Log10(TPOAb)	0.56 (0.37, 0.85)	0.007	0.93 (0.67, 1.29)	0.653
Log10(TgAb)	0.73 (0.53, 1.02)	0.063	0.99 (0.76, 1.29)	0.953

NOR, number of oocytes retrieved; FR, fertilization rate; TPOAb: thyroperoxidase antibody; TgAb, thyroglobulin antibody; BMI, body mass index; TSH, thyrotropin; FT3, free triiodothyronine; FT4, free thyroxine; Gn, gonadotropin; GnRH-a, gonadotropin-releasing hormone agonist; AFC, antral follicle count; ART, assisted reproductive technology; aOR, adjusted odds ratio; CI, confidence interval.

a Adjusted for maternal age; maternal BMI; TSH, FT3, and FT4 concentrations; total Gn dose; GnRH-a used; type of infertility; and AFC.

b Adjusted for maternal age; maternal BMI; TSH, FT3, and FT4 concentrations; total Gn dose; GnRH-a used; type of infertility; AFC; ART type used; and male age.

## Discussion

Our findings demonstrate a negative association between TAI and both the NOR and EQ in euthyroid women with infertility and DOR. Interestingly, we observed strong correlations of TAI with a decreased NOR or poor EQ only in the subgroup of women with a TSH concentration less than 2.5 mIU/L. Furthermore, higher TPOAb and TgAb concentrations were found to be associated with a lower NOR and EQ in this study population. The screening of women with POI and DOR for thyroid dysfunction (serum TSH) and thyroid antibodies should be performed in accordance with the recommendations made by the European Thyroid Association (ETA) in 2021 (32) and the European Society of Human Reproduction and Embryology (ESHRE) in 2016 (33). However, currently, there is very limited evidence regarding whether to treat TAI in DOR patients and the feasible effective treatment methods. These findings may support increased systematic screening to measure the TSH, TPOAb, and TgAb in infertile patients with DOR and timely treatment to improve fertility outcomes in this population.

Previous studies have mostly focused on investigating the impact of TAI on *in-vitro* ART outcomes on the overall population of women with infertility, with limited research conducted specifically on patients with DOR, and their results were controversial. There was no significant difference in EQ between women with TAI and those without TAI, as reported in both a large cohort of 3,444 euthyroid women in China (14) and a small cross-sectional analysis of infertile women (18). In contrast, in 2011, Monteleone et al. found that the FR (63% vs. 72%) and the rate of grade A embryos (25% vs. 48%) were significantly lower in patients with TAI than in patients without TAI (9). It has been hypothesized that TAI has a direct cytotoxic effect on oocytes and EQ (7, 9, 34). This assumption is supported by findings from other studies, as well as reports indicating that women with TAI have a lower FR and EQ than women without TAI (7, 9, 10). The reasons for the discrepancies in the results from previous research may be due to differences in population selection, reference value ranges, and statistical methods between studies. Compared with natural conception, ART serves as an ideal model for analyzing the impacts of various stages of the reproductive process. Research on ART involves numerous variables such as age, causes of infertility, ovarian stimulation protocols, types of ART treatments, thyroid antibody concentrations, and thyroid function. These variables make it difficult to attribute adverse outcomes solely to TAI (17). For example, the NOR is influenced by various parameters, including ovarian reserve, total Gn dosage, cause of infertility, and female age (35). In our study, we only included DOR patients with normal thyroid function and excluded patients receiving levothyroxine (LT4) treatment to eliminate potential influences from thyroid dysfunction. We observed a higher average age and a larger proportion of patients with a TSH exceeding 2.5 mIU/L in the TAI-positive group than in the TAI-negative group. Therefore, when investigating the association between TAI and NOR, we further adjusted our Poisson GLMs for potential confounders such as female age, BMI, type of infertility, AFC, thyroid function, total Gn dosage, and GnRH-a used. In addition, we further adjusted for the effects of the ART type used and male age on the EQ and FR in our GLMs. Our results suggested that TAI may be one of the risk factors for adverse reproductive outcomes in DOR patients. In addition, Safarian et al. found a strong positive association between the TPOAb concentrations in follicular fluid (FF) and serum and negative associations of the TPOAb concentration in FF with the NOR, AMH level, AFC, FR, and the number of high-quality embryos (7). Similarly, we found that higher serum TPOAb and TgAb concentrations were associated with a lower NOR and EQ. This might imply a potential dose-dependent effect of antibodies on the NOR and EQ.

A lower NOR and poorer EQ in women with TAI could be explained by various underlying mechanisms. Two additional studies have detected TPOAb and TgAb in human FF and reported correlations between the concentrations of these thyroid autoantibodies in FF and those in serum (7, 9). In addition, Lee et al. found that thyroid autoantibodies were present on the surface of embryos before transplantation and speculated that this affects embryonic development before implantation (36). Previous research has also proposed the full-effect or no-effect model, which suggests that the suppressive influence of TAI on the response to ovarian stimulation might depend upon the patient’s ovarian insufficiency status (37, 38). Recently, Huang et al. observed a chemokine inflammatory cascade in the environment surrounding the maturing oocyte in women with autoimmune thyroiditis (39). Specifically, the advancement of POI may be promoted by impaired follicular dynamics, which may be caused by enhanced cytotoxicity or a modified immunological condition associated with systemic inflammation (38). Therefore, TAI may negatively affect NOR by decreasing ovarian reserve (40, 41). Furthermore, as oocyte quality significantly influences the developmental competence of the embryo (42), any adverse impact on oocyte quality could consequently affect EQ.

Regarding the assessment of the potential for embryonic development, only one study on EQ in euthyroid women with DOR who were undergoing IVF treatment showed that EQ was impaired in women with both TPOAb positivity and TSH levels >2.5 mIU/L (16). It can be hypothesized that TAI, rather than the preconception of TSH concentration, affects the outcomes of IVF and ICSI cycles. TAI might reflect a reduced thyroid functional capacity (43), and during ovarian stimulation, women with TAI have increased TSH concentrations (44). Our study observed a higher proportion of women with TSH concentrations exceeding 2.5 mIU/L in the TAI-positive (vs. TAI-negative) group after excluding patients receiving LT4 treatment. Even after adjusting for thyroid function, however, TAI remained negatively correlated with the NOR and EQ in the subgroup of women with TSH concentrations less than 2.5 mIU/L, demonstrating the stability of our model. Our results suggest that TAI significantly affects the NOR and EQ in euthyroid women with DOR who are undergoing IVF or ICSI, consistent with the findings of Weghofer et al. (16). However, these results should be interpreted with caution and raise two possibilities. First, the sample of TAI-positive women in the TSH high-normal subgroup was small and thus may not have been sufficient to guarantee statistical significance. Second, compared with the slight increase in the TSH concentration in individuals with normal thyroid function, TAI may have more pronounced detrimental effects on the NOR and EQ. Therefore, further research is warranted to investigate the synergistic or additive effects of TAI and mild thyroid dysfunction on ovarian function decline.

However, no association was observed between TAI and FR in euthyroid patients with DOR. Our findings are aligned with those reported from studies of general populations of infertile women (14, 17, 37, 45) and are supported by the results of several meta-analyses (17, 45). However, two studies found lower FRs in women with TAI than in women without TAI (9, 46). The thyroid gland and zona pellucida have similar antigenic determinants; accordingly, thyroid antibodies can recognize and bind to the zona pellucida as a target, thus directly affecting the sperm–egg fusion (47). The ETA recommends that women with infertility and TAI should use ISCI (32). The suggestion that ICSI is more appropriate than other ART techniques for patients with TAI is predicated on the understanding that, compared with IVF, ICSI can surmount the suppressive effects of thyroid autoantibodies on sperm–egg fusion (9, 48). However, a retrospective study showed that in couples with non-male factor infertility, and TAI, there were no differences between the effects of ICSI and IVF on the EQ, CPR, and LBR (20). Although our study adjusted for male age and type of ART used when investigating the impact of TAI on the FR and EQ, TAI was not found to significantly affect the FR. The differences between the current study may be due to the influences of underlying male factors and sample size. Therefore, a large, directly comparative study is needed to evaluate the outcomes of ART in patients diagnosed with TAI who undergo either IVF or ICSI, with adjustments made for confounding factors such as the ages of both members of the couple, the cause of infertility, male parameters, and thyroid function.

We also found a correlation between TAI and an increased miscarriage rate, which is consistent with reduced EQ. As early as 2009, a study based on a TAI-positive mouse model showed that TPOAb binds to the preimplantation embryo, affecting its development after implantation and ultimately leading to fetal loss and reduced litter size (36). Research has indicated that in women with normal thyroid function, thyroid autoantibodies are associated with preterm birth (49–51) and miscarriage (6, 52, 53). However, a retrospective study found no impact of TAI on pregnancy outcomes in a population of 123 patients with DOR defined as FSH ≥12 IU/L (54). The possible reasons for the differences may lie in the different definitions of DOR and exclusion criteria. In comparison to their study, our study excluded patients with recurrent miscarriages. Additionally, differences in the adjustment of confounding factors may also contribute to the discrepancies in results: our study not only controlled for female age, BMI, TSH, and number and timing of transferred embryos but also further accounted for the impact of ART used and male age. Our research results provide further evidence to support systematic screening for TAI among women with DOR and subsequent treatment. LT4 treatment was recommended in subfertile women with TAI according to the 2021 ETA guideline’s recommendations (32). However, there is limited and controversial evidence regarding the use of LT4 therapy in infertile women with TAI (43, 55). Recent large-scale randomized controlled trials have shown that LT4 treatment did not improve pregnancy outcomes in patients with TAI (56, 57). These studies, which did not demonstrate the benefits of LT4, support the hypothesis that the increased risk of miscarriage in TAI patients is related to abnormal immune response rather than mild thyroid dysfunction. In a more recent study, it has been demonstrated that glucocorticoid may improve the pregnancy outcomes of ART women with TAI (58). More RCTs are needed to study the impact of immunotherapy on TAI in the future.

This study has several strengths. First, it is the first study to analyze the correlations between TAI, thyroid antibody concentrations, and the NOR, FR, and EQ in euthyroid women with DOR. Second, the research was conducted at a single center; accordingly, fertilization, *in-vitro* culture, and embryo scoring were carried out under identical settings, thereby reducing the potential for variability. However, this study also has certain limitations. First, it is a retrospective study using data from a single center, which may have introduced selection bias. In addition, differences in thyroid function during ovarian stimulation could not be assessed due to the retrospective nature of the study. Therefore, further prospective studies are needed to evaluate how variations in thyroid function might influence follicle development and EQ. Second, the TAI-positive group comprised a small proportion of the study population and had a relatively small sample size, which may have reduced the power of our statistical analysis. However, we used GLMs to adjust for confounding factors, including female age, male age, BMI, thyroid function, type of infertility, ART type used, total Gn dose, and GnRH-a used. This approach might have overcome discrepancies in the baseline patient characteristics between the two groups to further illustrate the link between TAI and the earliest stage outcomes of ART in euthyroid patients with infertility and DOR. Finally, this study did not address the impact of LT4 supplementation on women with TAI and DOR. Although patients using LT4 supplementation were excluded from our analysis, the number of subjects taking these supplements was less than 10% of the total study population, and their exclusion did not alter the results.

## Conclusion

Our results demonstrate a negative association between TAI and the *in-vitro* outcomes of ART, including the NOR and EQ, in patients with DOR. In women with TSH concentrations ≤2.5 mIU/L, TAI negatively affected the EQ. Furthermore, higher thyroid antibody concentrations were associated with a lower NOR and EQ in euthyroid women with DOR. Our findings provide patients and clinicians with further evidence suggesting that euthyroid patients with infertility and DOR should undergo screening for thyroid autoantibodies and treatment. In the future, well-designed prospective studies can evaluate whether LT4 supplementation may improve the *in-vitro* outcomes of ART in patients with DOR who are attempting to become pregnant. Additional large-sample randomized controlled trials are needed to further explore the impact of changes in the TSH concentration after ovarian stimulation on TAI and ART outcomes, and basic research is needed to explore the potential mechanisms underlying these associations.

## Data availability statement

The original contributions presented in the study are included in the article/**Supplementary Material**. Further inquiries can be directed to the corresponding author.

## Ethics statement

This study was reviewed by the Institutional Review Board and approved by the Ethics Committee of The Third Affiliated Hospital of Zhengzhou University (approval number: 2023–196-01). Written informed consent for participation was not required for this study in accordance with the national legislation and institutional requirements.

## Author contributions

YJZ: Conceptualization, Data curation, Methodology, Writing – original draft. YCZ: Conceptualization, Data curation, Formal analysis, Methodology, Writing – review & editing. ZS: Data curation, Writing – original draft. BR: Software, Writing – original draft. SY: Software, Writing – original draft. NX: Software, Writing – original draft. WL: Software, Writing – original draft. HL: Data curation, Formal analysis, Methodology, Resources, Supervision, Writing – review & editing.
