# Supplementary material for: Impaired embryo development potential associated with thyroid autoimmunity in euthyroid infertile women with diminished ovarian reserve
Source: Front Endocrinol (Lausanne). 2024 Jun 14;15:1376179. doi: 10.3389/fendo.2024.1376179 (PMC11214279; doi:10.3389/fendo.2024.1376179)
Supplement: Supplementary file 1 [file Table_1.docx]

Supplementary Material

## Supplementary Table 1

**TABLE S1 Associations between thyroid autoimmunity and in vitro fertilization or intracytoplasmic sperm injection outcomes.**

| Outcomes | TAI-positive | TAI-negative | OR (95% CI) | *P*-*adj** | *P* |
| --- | --- | --- | --- | --- | --- |
| Cycles with embryo transfer, n | 36 | 218 |  |  |  |
| Day of embryo transfer, n (%) |  |  | / | / | 0.535 |
| Cleavage | 28 (77.78) | 179 (82.11) |  |  |  |
| Blastocyst | 8 (22.22) | 39 (17.89) |  |  |  |
| No. of transferred embryos, n (%) |  |  | / | / | 0.383 |
| 1 | 17(47.22) | 120 (55.05) |  |  |  |
| 2 | 19 (52.78) | 98 (44.95) |  |  |  |
| CPR ^a^, n (%) | 19/36 (52.78) | 110/218 (50.46) | 1.12 (0.53, 2.38) | 0.768 | 0.797 |
| MR ^b^, n (%) | 7/19 (36.84) | 11/110 (10.00) | 4.37 (1.25, 15.25) | 0.021 | 0.004 |
| LBR ^c^, n (%) | 12/36 (33.33) | 99/218 (45.41) | 0.62 (0.28, 1.34) | 0.220 | 0.179 |

**Notes:** Binary logistic regression models were used for the data analysis. The models used to analyze the outcomes of ART were adjusted for maternal age; maternal BMI; TSH, FT3, and FT4 concentrations; total Gn dose; GnRH-a used; type of infertility; AFC; ART type used; day of embryo transfer; and number of transferred embryos; ^a^Clinical pregnancy was defined as at least one gestational sac in the uterus at 35 days after embryo transfer, as identified on ultrasonography; ^b^Miscarriage was defined as loss of clinical pregnancy before 28 weeks gestation; ^c^Live birth was defined as the delivery of at least one survived newborn, irrespective of gestation duration; TAI: thyroid autoimmunity; BMI, body mass index; TSH, thyrotropin; FT3, free triiodothyronine; FT4, free thyroxine; Gn, gonadotropin; GnRH-a, gonadotropin releasing hormone agonist; AFC, antral follicle count; ART: assisted reproductive technology; OR, odds ratio; CI, confidence interval; CPR, clinical pregnancy rate; MR, miscarriage rate; LBR, live birth rate.
